# TGF-β inhibition restores hematopoiesis and immune balance via bone marrow EPCs in aplastic anemia

**DOI:** 10.1038/s12276-025-01483-4

**Published:** 2025-06-30

**Authors:** Xin-Yan Zhang, Li-Ping Guo, Ya-Zhe Wang, Jin-Song Jia, Mi Liang, Meng-Zhu Shen, Zhen-Kun Wang, Zhi-Wei Zhang, Chen-Yuan Li, Zhong-Shi Lyu, Tong Xing, Yuan-Yuan Zhang, Xiao-Jun Huang, Yuan Kong

**Affiliations:** 1 Peking University People’s Hospital, Peking University Institute of Hematology, National Clinical Research Center for Hematologic Disease, Beijing Key Laboratory of Hematopoietic Stem Cell Transplantation, Collaborative Innovation Center of Hematology, Peking University, Beijing, China; 2 Peking-Tsinghua Center for Life Sciences, Academy for Advanced Interdisciplinary Studies, Peking University, Beijing, China

**Keywords:** Experimental models of disease, Translational research

## Abstract

Aplastic anemia (AA) is a life-threatening bone marrow (BM) failure syndrome characterized by pancytopenia. Recent studies revealed that dysfunctional endothelial progenitor cells (EPCs), critical components of the BM microenvironment, are involved in hematopoietic-dysfunction-related diseases, including AA. However, the mechanism underlying EPC damage in AA remains unknown. Here we find that transforming growth factor-β (TGF-β) signaling is hyperactive in dysfunctional AA EPCs with impaired hematopoietic support and immune regulatory ability, and TGF-β inhibition promotes hematopoiesis and immune rebalance by repairing dysfunctional EPCs. Through impaired EPC and AA murine models, we validated that TGF-β inhibition restores EPC dysfunction to improve hematopoiesis and immune status in vitro and in vivo. RNA sequencing and real-time quantitative polymerase chain reaction provided further validation. These results indicate that dysfunctional BM EPCs with hyperactive TGF-β signaling are involved in AA. TGF-β inhibition promotes multilineage hematopoiesis recovery and immune balance by repairing dysfunctional EPCs, providing a potential therapeutic strategy for AA.

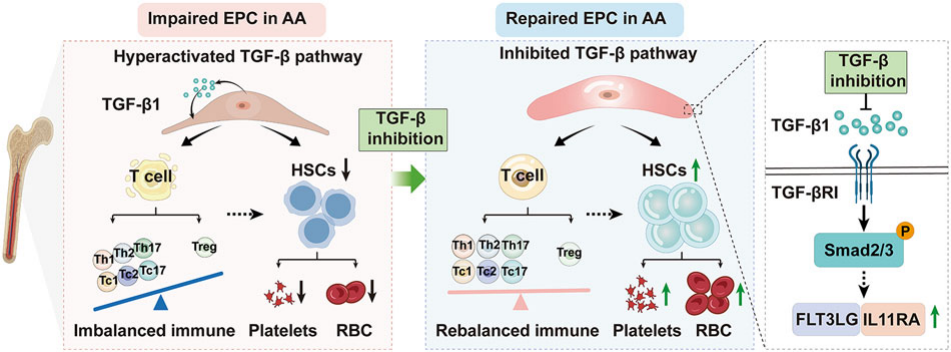

## TGF-β inhibition restores hematopoiesis and immune in aplastic anemia

Aplastic anemia is a serious condition where the bone marrow (BM) fails to produce enough blood cells. Researchers found that transforming growth factor-β (TGF-β) is overactive in BM endothelial progenitor cells (EPCs) of these patients, leading to impaired BM EPC function and poor blood cell production. The study involved both human patients and mice models to understand this process better. Researchers used a TGF-β inhibitor, which is a substance that blocks the action of TGF-β, to see if it could improve BM EPC function. They found that inhibiting TGF-β helped restore the ability of BM EPCs to support blood cell production and regulate the immune system. This was tested through various laboratory methods, including cell cultures and animal studies. Results suggest that targeting TGF-β could be a promising treatment strategy for aplastic anemia, potentially improving patient outcomes by enhancing multilineage hematopoiesis recovery and immune balance.

This summary was initially drafted using artificial intelligence, then revised and fact-checked by the author.

## Introduction

Aplastic anemia (AA) is a rare but life-threatening disease characterized by peripheral pancytopenia due to multilineage hematopoietic deficiency^1,2^. Accumulating evidences suggest that the pathogenesis of AA is manifold and involves T-cell-mediated immune destruction^3,4^, intrinsic defects in hematopoietic stem cells (HSCs)^5^ and that an impaired bone marrow (BM) microenvironment contributes to diminished hematopoietic capacity^6^. Effective treatments for severe AA are immunosuppressive therapies but with variable responsiveness^7^ or allogenic HSC transplantation (allo-HSCT) but with age restrictions^8^. As a complement, in-depth exploration of BM microenvironment may be a potential direction for AA amelioration.

Endothelial progenitor cells (EPCs) are crucial components of the BM microenvironment and regulate HSC fate and immune balance under physiological conditions or after myeloablation^9–13^. Conditional deletion of EPC-specific receptor (vascular endothelial growth factor receptor 2, VEGFR2) prevented EPC regeneration and hematopoiesis reconstitution in irradiated mice^14^, whereas infusion of EPCs can improve HSC and immune reconstitution in vivo^12,15,16^, which indicates that BM EPCs are essential to support hematopoiesis. EPCs also fulfill immunoregulation as a T cell mobilizer by production of chemokines^17^ or as novel innate immune cells with antigen presentation^18^. Moreover, dysregulated expansion of T helper 1 (T_H_1), T helper 2 (T_H_2) and T helper 17 (T_H_17) cells, whereas reduced or dysfunctional regulatory T cells (T_reg_) are consistent features of severe AA^4^. Expansion of B cells was also reported in patients with AA^19^. Mutually, anti-inflammatory angiogenic factors produced by EPCs can weaken T cell proliferation and alter T cell activation^20,21^. Recently, we reported that reactive oxygen species (ROS)-induced dysfunctional BM EPCs interfere with the self-renewal and differentiation of HSCs in multiple hematopoietic-dysfunction-related diseases, including corticosteroid-resistant immune thrombocytopenia, AA, myelodysplastic neoplasms (MDS) and poor hematopoietic reconstitution after allo-HSCT in vitro and in vivo^6,22–27^. Clinically, we conducted an open-label, randomized, phase III trial reinforced that hematopoietic reconstitution can be improved by repairing dysfunctional BM EPCs with *N*-acetyl-L-cysteine (a ROS scavenger) after allo-HSCT^28^. In addition, ROS-induced damage to the BM EPCs of mice and pateints with AA disabled not only their hematopoietic support function but also their immunoregulatory function^6^. However, how dysfunctional EPCs affect hematopoiesis and immunoregulation during the development of AA is not completely understood.

Transforming growth factor-β (TGF-β), an inflammatory cytokine produced primarily by HSC-adjacent niche cells such as Schwann cells and megakaryocytes attached around vessels, is an important component of the BM microenvironment^29–31^. Initially, TGF-β was considered a cardinal regulator that provides a quiescence signal to HSCs during homeostatic conditions^32–34^. However, accumulating evidences suggest that the effects of TGF-β on HSCs are more complex than previously thought. Different hematopoietic stem and progenitor cells (HSPC) respond differently to TGF-β1 in a stage-specific and dose-dependent manner^35–37^. High-dose TGF-β1 can arrest megakaryoblast maturation and erythroblast proliferation but augment lymphopoiesis^29,38,39^. Uniformly, the Smad2/3 circuitry downstream of TGF-β pathway is constitutively increased in erythroid progenitors of patients or disease models with ineffective erythropoiesis, such as MDS^40,41^ and β-thalassemia^42,43^. The clinical benefit of TGF-β superfamily ligand trap (luspatercept) for ineffective erythropoiesis is promising^44–48^. Moreover, recent research has confirmed that hyperactive TGF-β signaling in HSPCs contributes to BM failure in Fanconi anemia^49^ and Shwachman–Diamond syndrome^50^. Existing studies have primarily focused on the direct effects of TGF-β on hematopoietic cells, where it plays a crucial role in regulating HSCs by inducing quiescence and maintaining genomic stability^49–54^. However, its impact on EPC-mediated hematopoiesis has not been investigated in patients with BM disorders. Even though reports implicated that TGF-β induces apoptosis and prevents the proliferation of endothelial cells (ECs)^55,56^. Moreover, the role of TGF-β signaling in ECs is heterogeneous and pleiotropic^57–59^. TGF-β signaling either stimulates vascular formation or hampers biological function of vascular ECs, especially in their abilities of proliferation, migration and vessel maturation^59,60^. Thus, it raised our interest in whether activation of TGF-β signaling pathway in BM EPCs would perturb their regulation of hematopoietic support and immune balance in patients with AA.

In this study, we found that Smad2/3 signaling occurred in response to increased levels of TGF-β1 binding with type I TGFβ receptor (TGF-βRI) in BM EPCs from patients with AA compared with those from normal controls (NCs), which not only impeded erythropoiesis, megakaryopoiesis and myelopoiesis but also disrupted T cell differentiation. Furthermore, the hematopoiesis-supporting ability and immune-modulation function of BM EPCs were restored after TGF-β inhibition in vitro and in vivo. Together, these data suggest that TGF-β inhibition might be a promising approach for improving the prognosis of patients with AA.

## Materials and methods

### Patients and controls

Patients with AA (*N* = 15, aged 21–61 years with a median age of 54 years) and age-matched NCs (*N* = 15, aged 24–64 years with a median age of 53 years) were enrolled in this prospective case‒control study. There were no significant differences between the ages of patients with AA and NCs (Supplementary Table 1).

The Ethics Committee of Peking University People’s Hospital approved the study (2022PHB037-001), and all the subjects signed written informed consent in accordance with the Declaration of Helsinki.

### Isolation, cultivation and characterization of human primary BM EPCs

As previously reported^6,22–27^, BM mononuclear cells (BMMNCs) were separated with lymphocyte separation medium (GE Healthcare) and cultivated for 7 days. The identification of BM EPCs was performed with mouse anti-human CD45 (BD Biosciences), CD34 (BioLegend), VEGFR2 (CD309) (BD Biosciences) and CD133 (Miltenyi Biotec) monoclonal antibodies through flow cytometry via BD LSRFortessa (Becton Dickinson).

### Measurement of the levels of TGF-β1, TGF-βRI and p-Smad2/3 and the apoptosis ratio in BM EPCs and T cell subsets

After isolation from BMMNCs, the protein levels of TGF-β1 (BD Biosciences), TGF-βRI (Abcam) and phospho-Smad 2/3 (p-Smad2/3) (BD Biosciences) in BM EPCs were evaluated via flow cytometry. The cultivated BM EPCs were incubated with Annexin-V (BioLegend) and 7-amino-actinomycin D (7-AAD; BD Biosciences) to assess the apoptosis ratio of BM EPCs, which was measured via flow cytometry. As previously reported^22,24,61,62^, the apoptosis ratio includes the total percentage of early apoptosis cells(AnnexinV^+^7-AAD^−^) and late apoptosis cells (AnnexinV^+^7-AAD^+^), while live cells were defined as AnnexinV^−^7-AAD^−^. After 3 days of coculture, CD3^+^ T cells were collected and stimulated with a cocktail (eBioscience). The frequencies of different types of T cell were evaluated as previously reported^6,25^.

### Tube formation, migration and double-positive staining assays

The functions of EPCs were evaluated through tube formation, migration and double-positive staining assays following the published protocol^6,22–27,34^. After 7 days of cultivation, 4 × 10^4^ BM EPCs were seeded in Matrigel-coated (Corning) plates for 24 h. As previously reported^6,22–24,26,27,34,63–67^, tube formation was observed using a phase contrast microscope (Olympus). Three random fields were photographed in each group. The capillary tube length was calculated by drawing a line along each tube and measuring the length of the line in pixels using the ImageJ software. A total of 1 × 10^5^ BM EPCs were placed in the upper chambers (Corning) for 24 h to evaluate the number of migrated cells. A total of 1 × 10^5^ BM EPCs were stained with both diI-acetylated low-density lipoprotein (DiI-Ac-LDL) (Life Technologies) and fluorescein isothiocyanate-labeled UEA-1 (FITC-UEA-1) (Sigma) to evaluate the number of double-positive BM EPCs. All the cells were counted in three random fields.

### Coculture assays of EPC–HSC and EPC–T cell

The BM CD34^+^ cells were isolated from NC BMMNCs with CD34^+^ MicroBead kits (Miltenyi Biotec)^6,22–27,68^. The isolated HSCs derived from NCs (1 × 10^5^ per well) were cocultured with precultivated BM EPCs in StemSpan Serum-Free Expansion Medium (Stem Cell Technologies) for 5 days (Supplementary Fig. 1a). In addition, the BM CD3^+^ cells sorted from NC BMMNCs via CD3^+^ MicroBead kitsc (Miltenyi Biotec) were cocultured directly with precultivated BM EPCs in RPMI 1640 medium (Gibco) supplemented with 10% fetal bovine serum (Gibco) for 3 days^6,25^.

### EdU assay

After the coculture experiment of HSCs with BM EPCs, the HSCs were separately incubated with 50 μM 5-ethynyl-20-deoxyuridine (EdU) (RiboBio) for 24 h. The nuclear fluorescence intensity of the HSCs was evaluated via flow cytometry to investigate their proliferation ability of HSCs^25,69^.

### CFU assays

The hematopoiesis-supporting ability of EPCs was assessed via colony-forming unit (CFU) assays. A total of 1 × 10^5^ BM CD34^+^ cells from NCs were sorted via CD34^+^ microbead kits (Miltenyi Biotec) and then were performed direct-coculture assay with primary EPCs from patients with AA or NCs. After 5 days, the HSCs were collected and seeded in a 24-well plate (Corning) using MethoCult H4434 Classic (Stem Cell Technologies) for 14 days. The CFU erythroid (CFU-E), burst-forming unit erythroid (BFU-E), CFU granulocyte–macrophage (CFU-GM) and CFU granulocyte, erythroid, macrophage and megakaryocyte (CFU-GEMM) were scored as previously described^6,22–24,26,27^.

### Establishment of a BM EPC damage model in vitro

To establish the BM EPC damage model, the primary BM EPCs derived from NCs were treated with TGF-β1 (4 nM ml^−1^, PeproTech) in vitro for 24 h (ref. ^70^). To further determine the effect of TGF-β pathway inhibition on damaged EPC function, the EPCs were treated with TGF-β1 plus the TGF-βRI inhibitor galunisertib (LY2157299, LY, 75 nM ml^−1^, Selleck)^71^.

### Establishment of a classical AA murine model

The classical AA murine model was established as previously reported^3,6,72,73^. Hybrid (BALB/cBy × C57BL/6(B6)) F1 (CByB6F1) mice were treated with 5 Gy total-body irradiation (TBI) and then intravenously injected with allogeneic lymph nodes (5 × 10^6^ per mouse) from B6 mice 4‒6 h later on day 0. The B6 and CByB6F1 mice between 8 and 10 weeks of age were purchased from Beijing Vitalstar Biotechnology and bred there. The animal studies were approved by the Ethics Committee of Peking University People’s Hospital (2022PHE008).

### Treatment of AA mice with a TGF-βRI inhibitor

The TBI group and TBI and lymph nodes infusion group were orally administered LY (100 mg kg^−1^ per day) on days 1, 3, 5, 7, 9, 11 and 12. An equal volume of dimethyl sulfoxide (Sigma-Aldrich) was given to the untreated group at the same time.

On day 13, retroorbital blood was collected from the mice and used to analyze the number of blood cells with an automated hematology analyzer (Sysmex Corporation). The mice were euthanized to collect BM cells and femur tissues on day 14. The femur tissues were fixed in 4% paraformaldehyde and then separately stained with hematoxylin‒eosin and the EC marker endomucin (Santa Cruz Biotechnology) for immunohistochemistry to assess the percentages of different types of BM vessel and BM hematopoietic volume. In addition, the BM cells were collected to determine the cell counts of hematopoietic cells per million cells in BM, frequencies of myeloid cells (CD45^+^Gr-1^+^), B cells (CD45^+^CD3^−^B220^+^) and T cells (CD45^+^CD3^+^B220^−^)(Supplementary Fig. 1f) and the percentages of different types of T cell in the murine BM through flow cytometry^26,27^.

### Bulk RNA sequencing and data analysis

The BM EPCs derived from patients with AA (*N* = 4) and NCs (*N* = 4) were subjected to RNA sequencing (RNA-seq) analysis. Next-generation RNA-seq libraries were generated with the NEBNext Ultra II RNA Library Prep Kit (NEB, E7770S). The paired-end sequencing was performed on a NovaSeq 6000 (Illumina), which produced between 28 and 35 million 150-bp paired-end reads per sample. The raw FASTQ files were processed according to a previously described method and with modifications^68,74–76^. After removing low-quality reads and adapter sequences, the remaining reads were quantified against the SAF genome index (GRCh38 reference) at the transcript level using Salmon software in mapping-based mode and aggregated at the gene level using the R package tximport. The count values were calculated for differentially expressed genes (DEGs) with the R packages DESeq2 and IHW. The DEGs with weighted *P* values <0.05 and the absolute of a fold change(log_2_) >0.5 were used for Kyoto Encyclopedia of Genes and Genomes (KEGG) enrichment analysis using R package clusterProfiler.

### qRT‒PCR

The mRNA levels of *FLT3LG*, *KIT*, *FLT3* and *IL11RA* were detected with a SYBR-Green real-time quantitative polymerase chain reaction (qRT‒PCR) kit (Thermo Fisher Scientific). The 18S rRNA gene expression levels were used for normalization. All sequences of the primers used in this study are listed in Supplementary Table 2.

### Statistical analysis

The study data were analyzed with GraphPad Prism 8.0 (GraphPad). For the matched statistical data, the Wilcoxon matched-pairs signed rank test was used. For continuous data, the Mann‒Whitney *U* test and unpaired Student’s *t*-test were used. The results are presented as the means ± standard error of the mean (s.e.m.), and a *P* < 0.05 was considered statistically significant.

## Results

### TGF-β signaling pathway is hyperactive in impaired BM EPCs from patients with AA

**Fig. 1:**
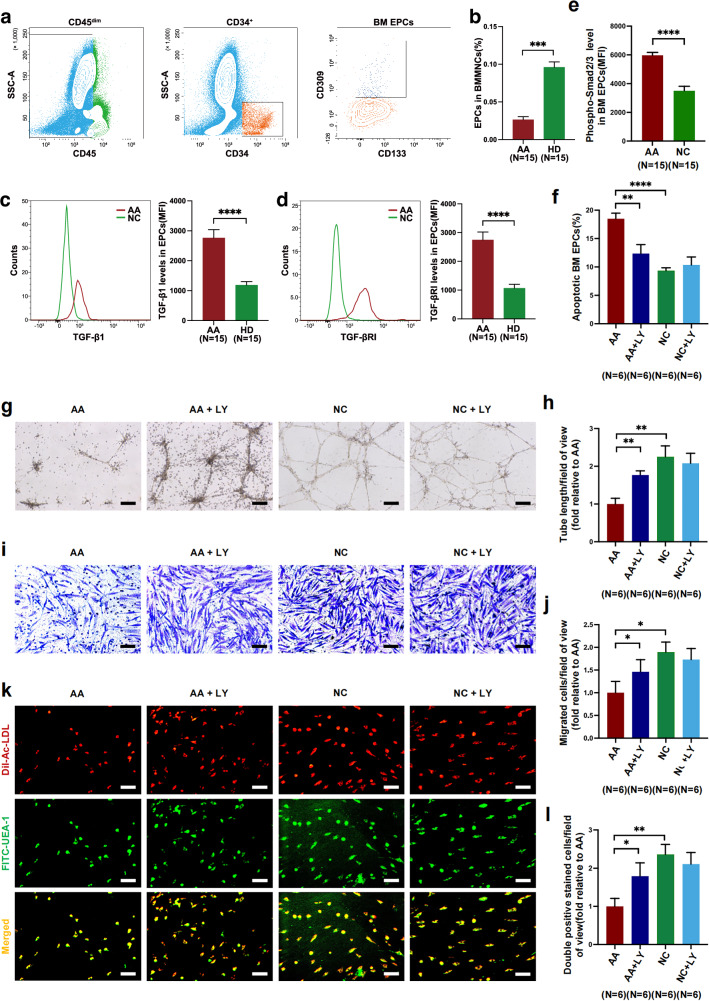
The levels of TGF-β1 and TGFβ-RI were higher in BM EPCs from patients with AA (AA EPCs) than in those from NC EPCs. **a**,**b**, Representative images (**a**) and the frequency (**b**) of BM EPCs (CD34^+^CD309^+^CD133^+^) among BMMNCs in AA EPCs and NC EPCs. **c**, A representative image showing the mean fluorescence intensity (MFI) of the TGF-β1 level and the expression level of TGF-β1 in AA EPCs and NC EPCs**. d**, A representative image of the TGFβ-RI level (MFI) and the expression level of TGFβ-RI in AA EPCs and NC EPCs. **e**, The expression level of p-Smad2/3 (MFI) in AA EPCs and NC EPCs. **f**, The apoptosis of AA EPCs and NC EPCs with or without TGF-β inhibition. **g**,**h**, Representative images (**g**) and quantification (**h**) of tube formation (pixels of tubes per field of view) by BM EPCs. Scale bars, 50 μm; original magnification, 10×. **i**,**j** Representative images (**i**) and quantification (**j**) of the transwell migration assay of cultivated BM EPCs. Scale bars, 50 μm; original magnification, 10×. **k**,**l**, Representative images (**k**) and quantification (**l**) of double-positive staining (merged in yellow) with DiI-Ac-LDL (red) and FITC-UEA-1 (green). Scale bars, 50 μm; original magnification, 10×. The data are presented as the mean ± s.e.m. (*P < 0.05, **P < 0.01, ***P < 0.001, ****P < 0.0001).

To investigate whether TGF-β signaling pathway is activated in BM EPCs in patients with AA, the expression levels of TGF-β1 and TGF-βRI in BM EPCs were compared through a prospective case‒control study involving AA patients and their age-matched NCs. The percentage of AA EPCs was significantly lower than that of NC EPCs (0.03 ± 0.004 versus 0.10 ± 0.007, *P* < 0.0001) (Fig. 1a,b). The expressions of TGF-β1 (2,762.00 ± 275.20 versus 1,186.00 ± 119.90, *P* < 0.0001) (Fig. 1c), TGF-βRI (2,742.00 ± 277.60 versus 1,067.00 ± 133.70, *P* < 0.0001) (Fig. 1d) and p-Smad2/3 (5,972.00 ± 200.80 versus 3,501.00 ± 315.40, *P* < 0.0001) (Fig. 1e and Supplementary Fig. 1b) in AA EPCs were significantly higher than those in NC EPCs. The primary EPCs from patients with AA or NCs were treated with or without TGF-βRI kinase inhibitor (galunisertib(LY2157299, hereafter referred to as LY)) for 24 h. Compared with NC EPCs, AA EPCs demonstrated functional damage characterized by an increased apoptosis ratio (18.48 ± 0.99 versus 9.68 ± 0.75, *P* < 0.0001) (Fig. 1f and Supplementary Fig. 1c), reduced tube formation (1.00 ± 0.15-fold versus 2.25 ± 0.29-fold, *P* = 0.009) (Fig. 1g,h) and cell migration (1.00 ± 0.25-fold versus 1.90 ± 0.22-fold, *P* = 0.04) (Fig. 1i,j) and decreased numbers of double-positive cells stained with DiI-Ac-LDL and FITC-UEA-1 (typical EPC markers; 1.00 ± 0.21-fold versus 2.36 ± 0.26-fold, *P* = 0.004) (Fig. 1k,l). Taken together, these results indicate that hyperactivation of TGF-β signaling pathway with downstream Smad2/3 phosphorylation is involved in numerically and functionally impaired BM EPCs in patients with AA, with high apoptosis and decreased abilities of angiogenesis and migration.

### The TGF-β inhibitor restores the dysfunction of AA EPCs in vitro

To determine whether hyperactive TGF-β signaling pathway is involved in the dysfunction of AA EPCs, we next examined whether the dysfunction of AA EPCs could be reversed by TGF-β inhibition. Compared with AA EPCs, LY treatment significantly reduced the degree of apoptosis (12.35 ± 1.59 versus 18.48 ± 0.99, *P* = 0.005) (Fig. 1f and Supplementary Fig. 1c), improved tube formation (1.77 ± 0.11-fold versus 1.00 ± 0.15-fold, *P* = 0.007) (Fig. 1g,h) and migration (1.46 ± 0.27-fold versus 1.00 ± 0.25-fold, *P* = 0.03) (Fig. 1i,j) and increased the number of double-positive stained cells (1.79 ± 0.35-fold versus 1.00 ± 0.21-fold, *P* = 0.02) (Fig. 1k,l). LY treatment had no significant effect on NC EPCs. These results suggest that TGF-β inhibition repairs the dysfunction of AA EPCs, resulting in reduced apoptosis and increased angiogenesis and migration in vitro.

### The TGF-β inhibitor ameliorates the hematopoiesis-supporting ability of AA EPCs in vitro

**Fig. 2:**
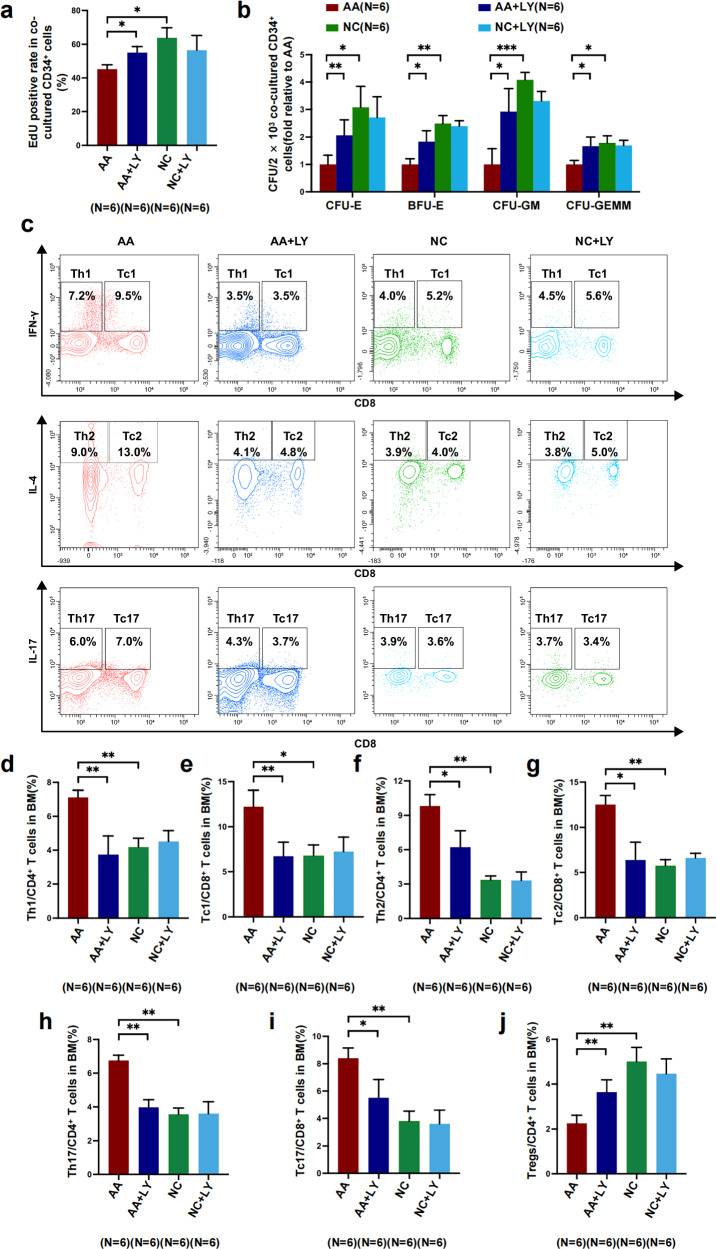
The abnormal hematopoiesis-supporting ability and immunomodulatory function of BM EPCs from patients with AA can be improved by inhibiting TGF-β pathway via in vitro coculture systems. **a**,**b**, The HSC EdU-positive ratio (**a**) and the CFU plating efficiency (**b**) of BM CD34^+^ cells from NCs after 5 days of coculture with cultivated AA EPCs or NC EPCs. **c**, Representative gating strategies of T_H_1 (CD3^+^CD8^-^IFN-γ^+^), T_H_2(CD3^+^CD8^-^IL-4^+^), and T_H_17 (CD3^+^CD8^-^IL^-^17^+^) cells among CD4^+^ T cells, as well as T_C_ cells 1(CD3^+^CD8^+^IFN-γ^+^), T_C_2 (CD3^+^CD8^+^IL-4^+^), and T_C_17 (CD3^+^CD8^+^IL-17^+^) cells among CD8^+^ T cells via flow cytometry, after 3 days of coculture with AA EPCs or NC EPCs with or without TGF-βRI kinase inhibitor. **d**–**j**, The frequencies of BM T_H_1 cells among CD4^+^ T cells (**d**), T_C_1 cells among CD8^+^ T cells (**e**), T_H_2 cells among CD4^+^ T cells (**f**), T_C_2 cells among CD8^+^ T cells (**g**), T_H_17 cells among CD4^+^ T cells (**h**), T_C_17 cells among CD8^+^ T cells (**i**) and T_reg_ (CD3^+^CD8^−^CD25^+^Foxp3^+^) among CD4^+^ T cells (**j**).

To evaluate whether inhibition of TGF-β signaling pathway could improve the hematopoiesis-supporting ability of dysfunctional EPCs, direct-contact coculture experiments of HSCs with AA EPCs or NC EPCs were performed to evaluate the proliferation and functions of HSCs after coculture. Compared with the group cocultured with NC EPCs, the AA group demonstrated worse hematopoiesis-supporting ability, characterized by a decreased ratio of EdU-positive HSCs (45.27 ± 2.60% versus 63.88 ± 6.01%, *P* = 0.02) (Fig. 2a) and worse CFU efficiencies, including CFU-E (1.00 ± 0.34-fold versus 3.08 ± 0.77-fold, *P* = 0.02) (Fig. 2b), BFU-E (1.00 ± 0.21-fold versus 2.48 ± 0.30-fold, *P* = 0.004) (Fig. 2b), CFU-GM (1.00 ± 0.58-fold versus 4.08 ± 0.28-fold, *P* = 0.0007) (Fig. 2b) and CFU-GEMM (1.00 ± 0.15-fold versus 1.79 ± 0.26-fold, *P* = 0.02) (Fig. 2b).

LY treatment improved the proliferation and function of HSCs in AA group, as shown by the increased ratio of EdU-positive HSCs (55.08 ± 3.57 versus 45.27 ± 2.60, *P* = 0.02) (Fig. 2a) and increased CFU efficiencies, including improved CFU-E (2.06 ± 0.56-fold versus 1.00 ± 0.34-fold, *P* = 0.008) (Fig. 2b), BFU-E (1.83 ± 0.39-fold versus 1.00 ± 0.21-fold, *P* = 0.01) (Fig. 2b), CFU-GM (2.92 ± 0.84-fold versus 1.00 ± 0.58-fold, *P* = 0.02) (Fig. 2b) and CFU-GEMM (1.66 ± 0.34-fold versus 1.00 ± 0.15-fold, *P* = 0.03) (Fig. 2b), whereas LY had no effect on the HSC-supporting ability of NC EPCs. These results indicate that the impaired hematopoiesis-supporting ability of AA EPCs could be restored by TGF-β inhibition.

### The TGF-β inhibitor rebalances the abnormal T cell differentiation-supporting ability of AA EPCs in vitro

TGF-β is a regulatory cytokine with a pleiotropic function in immune homeostasis and with context-dependent nature, including target cell type and cytokine milieu. As previously reported, the effects of TGF-β inhibition on T cells varied depending on downstream signals, resulting in either immune overactivation through uncontrolled activation of T cells and T_H_1 differentiation^77^ or immunosuppression through the reduction of T_H_17 differentiation and the enhancement of T_reg_ generation^78,79^. To explore the effect of TGF-βRI inhibition on the ability of BM EPCs modulating T cell differentiation, direct-contact coculture experiments of CD3^+^ T cells from NCs with AA EPCs or NC EPCs were performed. Representative flow cytometry gating strategies of T cell subsets after coculture are shown in Fig. 2c. Compared with the NC group, the AA group presented increased frequencies of T_H_1 (7.11 ± 0.43 versus 4.18 ± 0.53, *P* = 0.002) (Fig. 2d) and cytotoxic T type 1 (T_C_1) cells (12.22 ± 1.83 versus 6.78 ± 1.19, *P* = 0.03) (Fig. 2e), increased percentages of T_H_2 (9.82 ± 1.01 versus 3.36 ± 0.35, *P* = 0.002) (Fig. 2f) and cytotoxic T type 2 (T_C_2) cells (12.52 ± 1.02 versus 5.74 ± 0.67, *P* = 0.002) (Fig. 2g) and increased frequencies of T_H_17 (6.76 ± 0.31 versus 3.56 ± 0.38, *P* = 0.002) (Fig. 2h) and cytotoxic T type 17 (T_C_17) cells (8.41 ± 0.74 versus 3.82 ± 0.71, *P* = 0.002) (Fig. 2i) but a decreased frequency of T_reg_ (2.25 ± 0.35 versus 5.01 ± 0.63, *P* = 0.004) (Fig. 2j).

After LY treatment, AA EPCs were altered in their abilities to modulate T cell subset differentiation. LY treatment reduced the abnormal activation of three major immune types in AA EPC group, including the frequencies of T_H_1 (3.73 ± 1.11 versus 7.11 ± 0.43, *P* = 0.006) (Fig. 2d) and T_C_1 (6.71 ± 1.57 versus 12.22 ± 1.83, *P* = 0.002) (Fig. 2e) involved in type 1 immunity, the frequencies of T_H_2 (6.22 ± 1.44 versus 9.82 ± 1.01, *P* = 0.02) (Fig. 2f) and T_C_2 (6.38 ± 1.98 versus 12.52 ± 1.02, *P* = 0.02) (Fig. 2g) involved in type 2 immunity and the frequencies of T_H_17 (3.98 ± 0.45 versus 6.76 ± 0.31, *P* = 0.003) (Fig. 2h) and T_C_17 (5.51 ± 1.34 versus 8.41 ± 0.74, *P* = 0.01) (Fig. 2i) involved in type 3 immunity. Inversely, the frequency of T_reg_ increased after LY treatment in AA EPC group (3.64 ± 0.55 versus 2.25 ± 0.35, *P* = 0.008) (Fig. 2j). No significant differences were detected in the T cell differentiation-supporting ability of NC EPCs with or without LY.

In summary, we validated the abnormal expansion of the three major Th cells and reduction of T_reg_ in patients with AA in line with previous reports^4^. These data indicate that TGF-βRI inhibition on AA EPCs could attenuate the abnormal immune activation and restore T_reg_-mediated immune tolerance.

### Hyperactive TGF-β signaling pathway leads to BM EPC dysfunction, especially in terms of hematopoiesis and immunoregulation

**Fig. 3:**
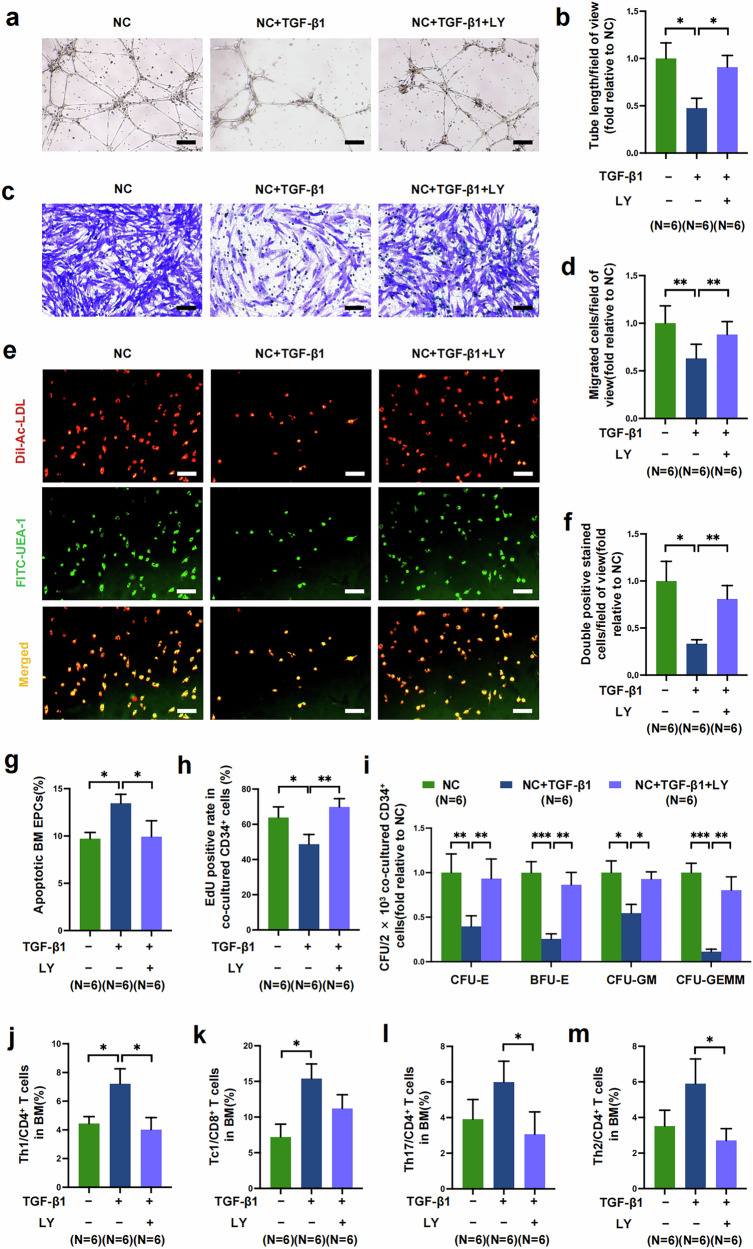
TGF-βRI kinase inhibitor improved the function of impaired EPCs triggered by TGF-β1 and reversed their impaired hematopoiesis-supporting ability and immunoregulation ability in vitro. **a**,**b**, Representative images (**a**) and quantification (**b**) of tube formation (pixels of tubes per field of view) of BM EPCs derived from NCs. Scale bars, 50 μm; original magnification, 10×. **c**,**d**, Representative images (**c**) and quantification (**d**) of the transwell migration assay for cultivated BM EPCs. Scale bars, 50 μm; original magnification, 10×. **e**,**f**, Representative images (**e**) and quantification (**f**) of the double-positive-stained cell assay for cultivated BM EPCs. **g**, The apoptotic ratio of cultivated BM EPCs. **h**,**i**, The EdU-positive ratio (**h**) and CFU plating efficiency (**i**) of BM CD34^+^ cells from NCs after 5 days of coculture with cultivated NC EPCs. **j**–**m**, The frequencies of T_H_1 cells among CD4^+^ T cells (**j**), and T_C_1 cells among CD8^+^ T cells (**k**), T_H_17 cells among CD4^+^ T cells (**l**) and T_H_2 cells among CD4^+^ T cells (**m**), after 3 days of coculture of BM CD3^+^ cells with NC EPCs. The data are presented as the mean ± s.e.m. (*P < 0.05, **P < 0.01, ***P < 0.001, ****P < 0.0001).

To further validate our hypothesis that hyperactive TGF-β signaling pathway may lead to BM EPC dysfunction, an in vitro model of TGF-β1-triggered BM EPC damage was established. BM EPC function significantly deteriorated after exogenous TGF-β1 exposure, as demonstrated by decreased tube formation (0.48 ± 0.10-fold versus 1.00 ± 0.16-fold, *P* = 0.03) (Fig. 3a,b) and migration abilities (0.63 ± 0.15-fold versus 1.00 ± 0.18-fold, *P* = 0.003) (Fig. 3c,d), as well as a reduced number of double-positive stained cells (0.33 ± 0.04-fold versus 1.00 ± 0.21-fold, *P* = 0.01) (Fig. 3e,f) but increased the apoptosis rate of BM EPCs (13.45 ± 0.95 versus 9.72 ± 0.66, *P* = 0.03) (Fig. 3g).

TGF-β1 treatment contributed to a decreased hematopoiesis-supporting ability of BM EPCs, including a lower EdU ratio of cocultured HSCs (48.72 ± 5.48 versus 63.88 ± 6.01, *P* = 0.01) (Fig. 3h) and decreased counts of CFU-E (0.40 ± 0.12-fold versus 1.00 ± 0.21-fold, *P* = 0.002) (Fig. 3i), BFU-E (0.25 ± 0.06-fold versus 1.00 ± 0.12-fold, *P* = 0.0003) (Fig. 3i), CFU-GM (0.55 ± 0.10-fold versus 1.00 ± 0.13-fold, *P* = 0.02) (Fig. 3i) and CFU-GEMM (0.11 ± 0.03-fold versus 1.00 ± 0.11-fold, *P* = 0.0003) (Fig. 3i), compared with those in NC group alone.

In terms of immunoregulation, the frequencies of T cell subsets were detected via flow cytometry after coculture with EPCs (Supplementary Fig. 1d). Type 1 T cells, including T_H_1 (7.21 ± 1.04 versus 4.44 ± 0.48, *P* = 0.03) (Fig. 3j) and T_C_1 cells (15.40 ± 2.05 versus 7.21 ± 1.79, *P* = 0.03) (Fig. 3k), were significantly increased in TGF-β1 group. These findings indicate that hyperactive TGF-β pathway contributes to dysfunctional BM EPCs with increased apoptosis, decreased angiogenesis, impaired hematopoiesis-supporting ability and impaired immunoregulation ability.

### The TGF-β inhibitor restores the impaired BM EPCs induced by TGF-β1 in vitro

Although hyperactive TGF-β1 led to BM EPC dysfunction, LY treatment improved tube formation (0.91 ± 0.12-fold versus 0.48 ± 0.10-fold, *P* = 0.02) (Fig. 3a,b) and migration (0.88 ± 0.14-fold versus 0.63 ± 0.15-fold, *P* = 0.006) (Fig. 3c,d), increased the number of double-positive stained EPCs (0.81 ± 0.14-fold versus 0.33 ± 0.04-fold, *P* = 0.007) (Fig. 3e,f) and reduced the apoptosis ratio of BM EPCs (9.93 ± 1.68 versus 13.45 ± 0.95, *P* = 0.03) (Fig. 3g).

LY treatment reversed the deteriorated hematopoiesis-supporting ability of BM EPCs caused by TGF-β1, as indicated by the increased EdU ratio of cocultured HSCs (69.85 ± 4.74 versus 48.72 ± 5.48, *P* = 0.004) (Fig. 3h) and improved CFU efficiencies, including CFU-E (0.93 ± 0.22-fold versus 0.40 ± 0.12-fold, *P* = 0.009) (Fig. 3i), BFU-E (0.86 ± 0.14-fold versus 0.25 ± 0.06-fold, *P* = 0.001) (Fig. 3i), CFU-GM (0.93 ± 0.08-fold versus 0.55 ± 0.10-fold, *P* = 0.03) (Fig. 3i) and CFU-GEMM (0.80 ± 0.15-fold versus 0.11 ± 0.03-fold, *P* = 0.005) (Fig. 3i). Compared with in coculture with TGF-β1-exposed EPCs, the differentiation potential of T cells was rebalanced when in coculture with TGF-β1-exposed EPCs and LY treatment, as indicated by decreased ratios of T_H_1 (4.02 ± 0.84 versus 7.21 ± 1.04, *P* = 0.03) (Figs. 3j), T_H_17 (3.06 ± 1.26 versus 5.99 ± 1.18, *P* = 0.03) (Fig. 3l) and T_H_2 cells (2.71 ± 0.66 versus 5.90 ± 1.39, *P* = 0.03) (Fig. 3m).

Consistent with the results of AA EPCs, the TGF-β1-exposed EPCs and LY treatment model also validated that the TGF-β inhibitor could repair dysfunctional BM EPCs, resulting in decreasing apoptosis, increasing angiogenesis and migration, and improving hematopoietic support and immunoregulation ability.

### AA mice exhibit decreased BM ECs and hyperactive TGF-β signaling pathway

**Fig. 4:**
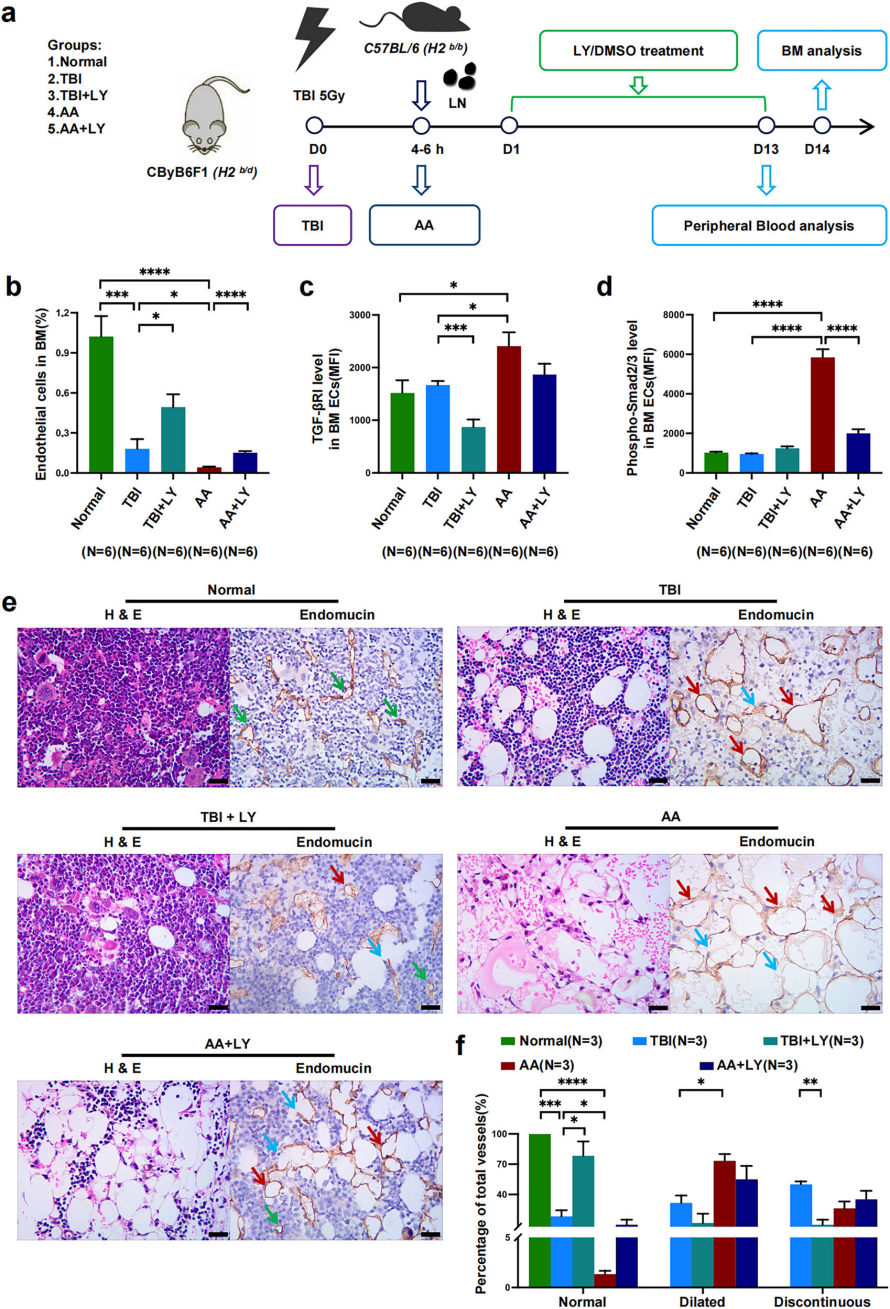
Decreased number of BM ECs with hyperactivation of TGF-β signaling pathway in AA mice. **a**, The establishment of classical AA mice with preirradiation and lymph node (LN) injection and schematic diagram of the different groups and treatments of the mice. **b**–**d**, The percentages of ECs (**b**) and the expression levels of TGFβ-RI (MFI) (**c**) and p-Smad2/3 (MFI) (**d**) in murine BM ECs. **e**, Representative images of BM vessels stained with an anti-endomucin antibody (scale bars, 10 μm) and BM sections stained with hematoxylin‒eosin (scale bars, 10 μm) in murine femurs. The green arrows represent normal vessels, the red arrows represent dilated vessels and the blue arrows represent discontinuous vessels in the murine femur. **f**, The percentages of normal and dilated as well as discontinuous BM vessels among the total vessels in the different murine groups. The data are presented as the mean ± s.e.m. (*P < 0.05, **P < 0.01, ***P < 0.001, ****P < 0.0001).

A classical AA murine model was established to further investigate whether TGF-β signaling is activated in impaired BM ECs in vivo (Fig. 4a). Compared with TBI mice, AA mice presented significantly fewer BM ECs (CD45^−^Ter119^−^CD31^+^VE-Cadherin^+^; 0.04 ± 0.01 versus 0.18 ± 0.07, *P* = 0.03) (Fig. 4b) but hyperactive expression of TGF-βRI (2,406.00 ± 264.30 versus 1,665.00 ± 78.71, *P* = 0.02) (Fig. 4c) and p-Smad2/3 in BM ECs (5,848.00 ± 408.30 versus 954.50 ± 37.42, *P* < 0.0001) (Fig. 4d). We next performed hematoxylin‒eosin staining and immunohistochemistry with anti-endomucin to evaluate the structure of BM vessels in the different groups (Fig. 4e). AA mice consistently presented a significantly disrupted BM vessel structure (normal: 1.33 ± 0.33 versus 18.33 ± 6.01, *P* = 0.05, dilated: 73.33 ± 6.67 versus 31.67 ± 7.27; *P* = 0.01) (Fig. 4f) compared with that of TBI mice. Therefore, these results further confirm our previous findings and indicate decreased and damaged BM ECs with hyperactive TGF-β pathway in AA mice.

### TGF-β pathway inhibition enhances the regeneration of BM ECs and vessels in AA mice

To further investigate the effect of TGF-βRI inhibitor on BM ECs in vivo, the classical AA murine model was treated with LY. After oral administration of LY for 13 days in AA mice (AA + LY mice), the ratio of BM ECs in AA + LY mice was markedly higher (0.15 ± 0.01 versus 0.04 ± 0.01, *P* < 0.0001) (Fig. 4b) than that in AA mice. The expression levels of TGF-βRI (1,869.00 ± 204.10 versus 2,406.00 ± 264.30) (Fig. 4c) and p-Smad2/3 (2,007.00 ± 206.10 versus 5,848.00 ± 408.30, *P* < 0.0001) (Fig. 4d) in BM ECs decreased in AA + LY mice than those in AA mice. In addition, higher percentages of normal BM vessels were detected in AA + LY mice (10.00 ± 5.00 versus 1.33 ± 0.33) (Fig. 4f) than in AA mice. Therefore, inhibition of TGF-β pathway promotes the repair of BM vessels and BM EC function.

### TGF-β pathway inhibition might promote hematopoietic recovery by repairing EC damage in AA mice

**Fig. 5:**
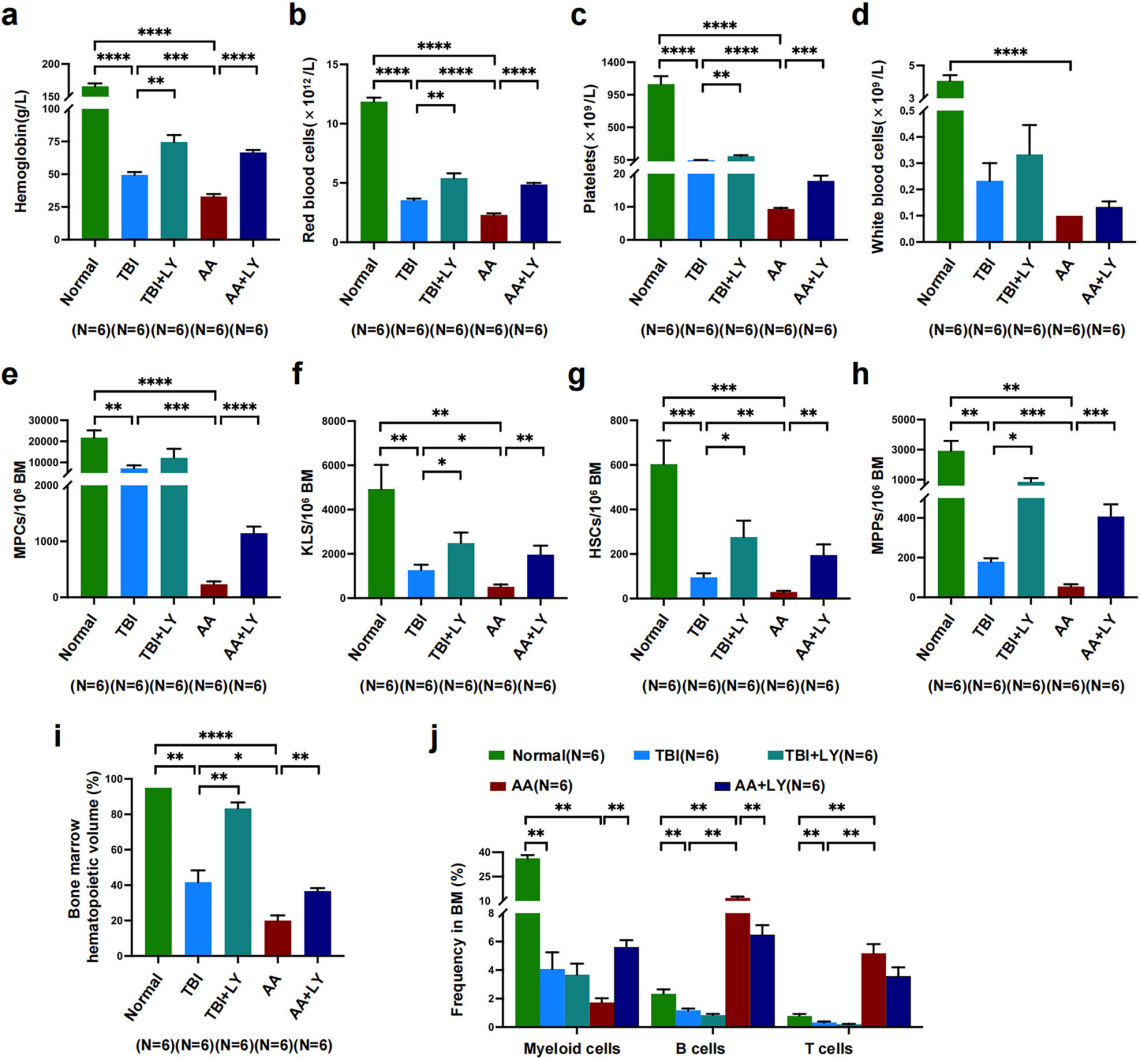
TGF-β inhibition improved hematopoietic recovery by repairing BM EC damage in AA mice. **a**–**d**, Counts of hemoglobin (**a**), red blood cells (RBCs) (**b**), platelets (**c**) and white blood cells (WBCs) (**d**) in all the mouse groups. **e**–**h**, The counts of myeloid progenitor cells (MPCs) (lineage^−^cKIT^+^SCA1^−^) (**e**), KLS (cKIT^+^lineage^−^SCA1^+^) cells (**f**), HSCs (**g**) and multipotent progenitors (MPPs) (lineage^−^SCA1^+^cKIT^+^CD150^−^CD48^−^) (**h**) per million cells within murine BM. **i**, The hematopoietic volume in murine BM. **j**, The frequencies of myeloid cells (CD45^+^Gr-1^+^), B cells (CD45^+^CD3^−^B220^+^) and T cells (CD45^+^CD3^+^B220^−^) among CD45^+^ cells in murine BM. The data are presented as the mean ± s.e.m. (*P < 0.05, **P < 0.01, ***P < 0.001, ****P < 0.0001).

To further validate whether inhibition of TGF-β pathway modulates hematopoietic recovery in vivo, peripheral blood cells and BM HSPCs were analyzed in AA mice. AA + LY mice presented better peripheral blood cell performance, including improved hemoglobin (66.67 ± 1.87 g l^−1^ versus 33 ± 1.90 g l^−1^, *P* < 0.0001) (Fig. 5a), red blood cell ((4.87 ± 0.14) × 10^12^ l^−1^ versus (2.30 ± 0.13) × 10^12^ l^−1^, *P* < 0.0001) (Fig. 5b), platelet ((17.83 ± 1.58) × 10^9^ l^−1^ versus (9.33 ± 0.33) × 10^9^ l^−1^, *P* = 0.0004) (Fig. 5c) and white blood cell ((0.13 ± 0.02) × 10^9^ l^−1^ versus (0.10 ± 0.00) × 10^9^ l^−1^) (Fig. 5d) counts.

The gating strategy for hematopietic cells is displayed in Supplementary Fig. 1e. Higher cell counts per million cells in BM of myeloid progenitor cells (lineage^−^cKIT^+^SCA1^−^; 1,145.00 ± 117.60 versus 233.40 ± 50.96, *P* < 0.0001) (Fig. 5e), KLS (cKIT^+^lineage^−^SCA1^+^) cells (1,953.00 ± 416.80 versus 512.30 ± 106.40, *P* = 0.007) (Fig. 5f), HSCs (lineage^−^cKIT^+^SCA1^+^CD150^+^CD48^−^; 195.10 ± 48.17 versus 28.93 ± 5.71, *P* = 0.007) (Fig. 5g) and multipotent progenitors (lineage^−^SCA1^+^cKIT^+^CD150^−^CD48^−^; 407.10 ± 61.51 versus 53.94 ± 11.97, *P* = 0.0002) (Fig. 5h) were found in AA + LY mice than in AA mice. In addition, a greater hematopoietic volume in BM was observed in AA + LY mice (36.67 ± 1.67 versus 20.00 ± 2.89, *P* = 0.008) (Fig. 5i) than in AA mice, which demonstrated that TGF-β pathway inhibition improved hematopoietic regeneration in AA mice.

The gating strategies for myeloid cells, B cells and T cells in murine BM are displayed in Supplementary Fig. 1f. AA + LY mice displayed improved hematopoietic lineage and myeloid cells (5.61 ± 0.48 versus 1.73 ± 0.29, *P* = 0.002) (Fig. 5j), whereas lower frequencies of B cells (6.49 ± 0.66 versus 11.82 ± 0.78, *P* = 0.002) (Fig. 5j) and T cells (3.59 ± 0.60 versus 5.18 ± 0.65) (Fig. 5j) in BM. Taken together, these findings indicate that inhibition of the hyperactive TGF-β pathway could improve multilineage hematopoiesis recovery and rebalance the immune homeostasis, which might be achieved by repairing BM EC damage in AA mice.

### TGF-β pathway inhibition regulates T cell differentiation in AA mice

**Fig. 6:**
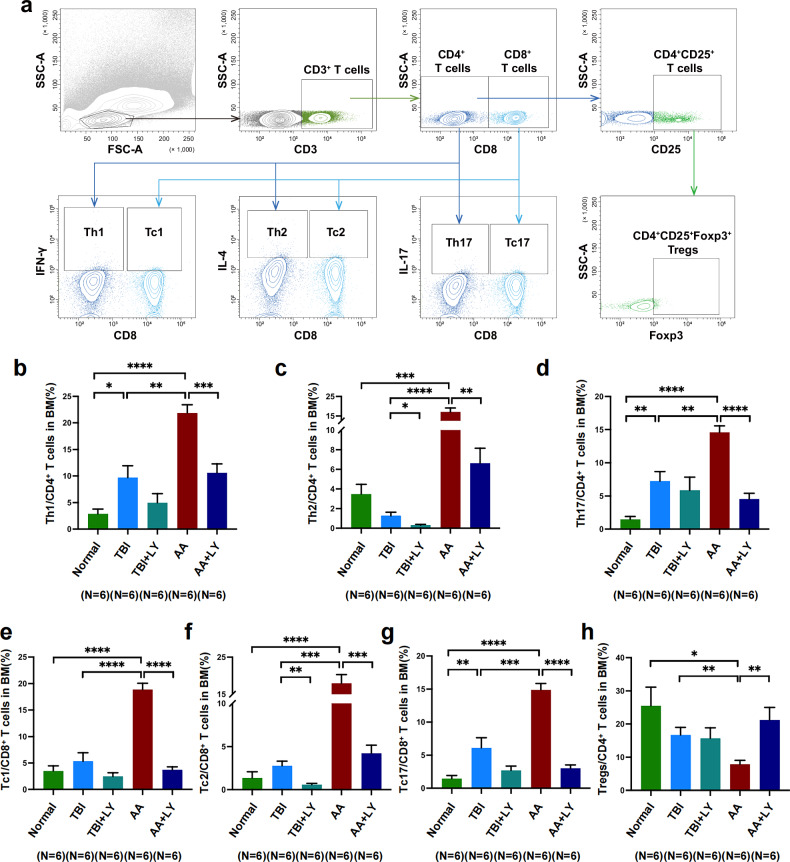
TGF-β inhibition improved the immunological status of AA mice. **a**, Representative flow cytometry gating strategies for murine BM T_H_1, T_H_2, T_H_17, T_C_1, T_C_2 and T_C_17 cells and T_reg_. **b**–**d**, The percentages of BM T_H_1 cells (**b**), T_H_2 cells (**c**) and T_H_17 cells (**d**) among BM CD4^+^ T cells in all the mouse groups. **e**–**g**, The percentages of BM T_C_1 cells (**e**), T_C_2 cells (**f**) and T_C_17 cells (**g**) among BM CD8^+^ T cells in all the mouse groups. **h**, The percentage of T_reg_ among BM CD4^+^ T cells in all the mouse groups. The data are presented as the mean ± s.e.m. (*P < 0.05, **P < 0.01, ***P < 0.001, ****P < 0.0001).

Since the in vitro findings suggested that TGF-β pathway inhibition in EPCs could regulate T cell differentiation in AA group, we investigated the effect of TGF-β inhibition in AA mice. The gating strategies for T_H_1, T_H_2, T_H_17, T_C_1, T_C_2 and T_C_17 cells and T_reg_ in BM are shown in Fig. 6a. The frequencies of T_H_1 (10.61 ± 1.68 versus 21.85 ± 1.57, *P* = 0.0006) (Fig. 6b), T_H_2 (6.63 ± 1.52 versus 17.08 ± 2.00, *P* = 0.002) (Fig. 6c), T_H_17 (4.54 ± 0.87 versus 14.62 ± 0.95, *P* < 0.0001) (Fig. 6d), T_C_1 (3.71 ± 0.56 versus 18.85 ± 1.20, *P* < 0.0001) (Fig. 6e), T_C_2 (4.23 ± 0.94 versus 17.94 ± 2.38, *P* = 0.0003) (Fig. 6f) and T_C_17 cells (3.02 ± 0.51 versus 14.88 ± 0.97, *P* < 0.0001) (Fig. 6g) were significantly decreased, but the frequency of T_reg_ was increased (21.21 ± 3.82 versus 7.90 ± 1.15, *P* = 0.008) (Fig. 6h) in AA mice administered with LY compared with untreated AA mice. The results from in vivo and in vitro coculture models uniformly demonstrated that TGF-β pathway inhibition could decrease aberrant immune activation and recover T_reg_-mediated immune tolerance by repairing BM ECs in AA mice.

### RNA-seq analysis validates that TGF-β signaling pathway is related to BM EPC dysfunction

**Fig. 7:**
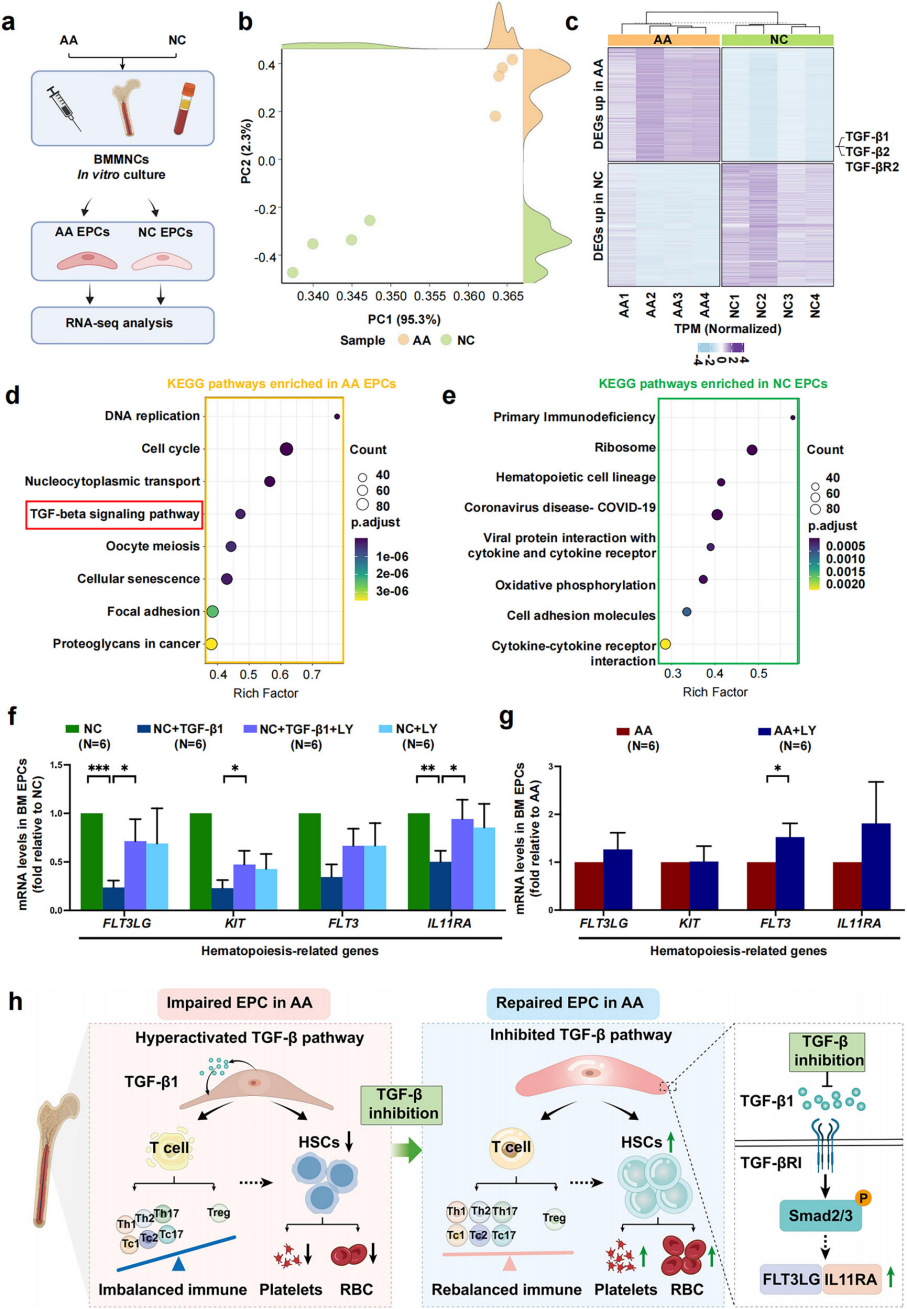
RNA-seq validated that TGF-β pathway is related to BM EPC dysfunction in patients with AA. **a**, A schematic diagram of the study design for RNA-seq analysis of AA EPCs and NC EPCs. **b**, A principal component analysis (PCA) of AA EPCs and NC EPCs. **c**, A heat map of upregulated and downregulated genes in AA EPCs and NC EPCs. **d**, A KEGG pathway analysis showing that TGF-β pathway was upregulated in AA EPCs. **e**, A KEGG analysis of enriched pathways in NC EPCs. **f**,**g**, The mRNA levels of hematopoietic-related genes in impaired EPC models (**f**) and in AA EPCs (**g**). **h**, A schematic illustration indicating that hyperactive TGF-β signaling pathway with downstream Smad2/3 phosphorylation contributes to dysfunctional BM EPCs in AA, whereas TGF-β inhibition repairs dysfunctional BM EPCs, decreasing apoptosis, increasing angiogenesis and migration, and improving multilineage hematopoiesis and immune balance in vitro and in vivo, providing a potential therapeutic strategy for patients with AA. The data are presented as the mean ± s.e.m. (*P < 0.05, **P < 0.01, ***P < 0.001, ****P < 0.0001).

To investigate the potential mechanism of BM EPC dysfunction in patients with AA, RNA-seq was performed on BM EPCs from four pairs of patients with AA and their age-matched NCs (Fig. 7a). A principal component analysis demonstrated that the expression patterns of EPCs in patients with AA and NCs were completely different (Fig. 7b). The expression levels of TGF-β1, TGF-β2 and type II TGF-β receptor were significantly higher in AA EPCs than in NC EPCs (Fig. 7c). The KEGG pathway gene set enrichment analysis showed that TGF-β signaling pathway were significantly upregulated in AA EPCs compared with NC EPCs (Fig. 7d). Besides, the pathways involved with the hematopoietic cell lineage commitment and differentiation and immune regulation were significantly downregulated in AA EPCs compared with NC EPCs (Fig. 7e), which verified the important role of EPCs in supporting hematopoiesis and regulating immunology at the genetic level.

The mRNA levels of hematopoiesis-related genes, including *FLT3LG* (0.24 ± 0.07 versus 1.00 ± 0.00, *P* = 0.0001) (Fig. 7f) and *IL11RA* (0.50 ± 0.11 versus 1.00 ± 0.00, *P* = 0.001) (Fig. 7f), were decreased in the impaired BM EPC model induced by TGF-β1, as detected via qRT‒PCR. After inhibition of TGF-β in the impaired EPC model, the expressions of genes, including *FLT3LG* (0.71 ± 0.23 versus 0.24 ± 0.07, *P* = 0.04) (Fig. 7f) and *IL11RA* (0.94 ± 0.20 versus 0.50 ± 0.11, *P* = 0.03) (Fig. 7g), were restored. After inhibition of TGF-β, a similar improvement in the mRNA levels of hematopoiesis-related genes, such as *FLT3* (3.38 ± 1.64 versus 1.00 ± 0.00, *P* = 0.03) (Fig. 7g) and *IL11RA* (1.81 ± 0.87 versus 1.00 ± 0.00) (Fig. 7g) was detected in BM EPCs from patients with AA, suggesting that these genes may be potential targets of TGF-β pathway that regulate hematopoiesis.

## Discussion

Although the involvement of dysfunctional EPCs in the development of AA was demonstrated in our previous work^6^, there is currently no tangible explanation for the underlying molecular mechanism involved. Here, we reverified that BM EPCs from patients with AA were numerically and functionally impaired and further revealed that overactivation of canonical TGF-β pathway, with downstream Smad2/3 phosphorylation contributing to increased apoptosis, decreased angiogenesis, impaired hematopoiesis support and imbalanced immunoregulation abilities in BM EPCs from patients with AA. TGF-β inhibition could restore the multilineage hematopoietic support and T cell immune rebalancing capacity of BM EPCs in vitro or in AA mice (Fig. 7h).

Hyperactive TGF-β signaling has been implicated in the pathogenesis of several anemia-associated diseases, such as MDS^47,48,80^, β-thalassemia^81–83^ and primary myelofibrosis^84^ and the prognosis of these diseases benefits from luspatercept, a TGF-β superfamily ligand trap that improves anemia by promoting late-stage erythropoiesis^82,85^. However, there are no reports of luspatercept in the treatment of AA. Intriguingly, patients with MDS were enrolled in the COMMANDS trial (NCT03682536), a phase III study that compared the efficacy and safety of luspatercept with those of epoetin alfa and achieved hematologic improvements in erythrocytes, neutrophils and platelets after receiving luspatercept^47,86^. Indeed, a recent study suggested that RAP-536, an analog of luspatercept, has no direct effect on HSPCs^80^. These findings indicate the potential role of luspatercept in the BM microenvironment.

In the current study, we demonstrate that TGF-β signaling is overactivated by autocrine secretion of TGF-β1 in BM EPCs of patients with AA and in classical AA mice, resulting in peripheral pancytopenia. In line with the multilineage hematopoietic restoration effects of luspatercept in patients with MDS^45,86^, we found that galunisertib (LY2157299 monohydrate), a TGF-βRI kinase inhibitor that downregulates the phosphorylation of Smad2/3, improved multilineage hematopoiesis in an AA mouse model. In addition, previous studies have shown that EPC or EC infusion can augment BM HSC reconstitution and the production of mature blood cells^15,87^. TGF-β inhibition not only repaired dysfunctional BM EPCs but also expanded the number of ECs endogenously, which could eliminate access restrictions associated with BM EPC or EC infusion.

With respect to the mechanism underlying TGF-β signaling-induced hematopoiesis damage, qRT‒PCR analysis revealed that TGF-β1 inhibited hematopoiesis gene expression in BM EPCs via *FLT3LG* and *IL11RA*, which could be reversed by LY. As previously reported, FLT3 is essential hematopoietic factor with prominent effects on the development of hematopoietic multilineages^88^, and IL11RA is the receptor for interleukin-11 (IL-11) which promotes HSC engraftment in AA mice via promoting megakaryopoiesis^89^. These results are consistent with our findings in vitro and in vivo, indicating the pivotal role of TGF-β signaling in regulating the hematopoiesis-supporting capacity of BM EPCs.

In addition to the indirect suppression of hematopoiesis by TGF-β-activated BM EPCs, direct regulation of TGF-β signaling on HSPCs cannot be excluded in the development of AA. During homeostasis, TGF-β was reported to directly maintain HSC hibernation through up-regulating p57 cyclin-dependent kinase inhibitor^51^. Beyond physiological effects, TGF-β directly causes HSPC growth arrest in mice and patients with Fanconi anemia^49^. However, maintenance of HSC quiescence is dependent on BM microenvironment, such as megakaryocyte-derived TGF-β rather than an autocrine TGF-β signaling loop in HSCs^32,51^. Although further validation in a BM EC-specific TGF-βRI-overexpressing murine model is needed, the present study is the first to demonstrate that LY2157299 monohydrate inhibits TGF-βRI kinase in impaired BM EPCs and restores hematopoiesis and immune homeostasis, identifying a potential mechanism in AA.

Different from other BM failure disease, AA has been well characterized as an immune-mediated BM disorder^1–4^. In our study, abnormal expansion of T_H_1, T_H_2, and T_H_17 cells and reduction of T_reg_ cells were observed in cocultures with AA EPCs, and the differentiation potential of T cells was rebalanced after LY treatment, confirming that TGF-β1-activated EPCs are defective in modulating immune homeostasis in AA. In addition to the abnormal number of T cell subsets, functional abnormalities of T_reg_ cells were also reported in AA^4,6,90^. However, we are aware that the function of rebalanced T cells in cocultures with AA EPCs needs to be further explored in the future.

Our findings show that dysfunctional BM EPCs with hyperactive TGF-β signaling are involved in AA, whereas the inhibition of TGF-β pathway could promote multilineage hematopoiesis recovery and immune balance in AA via the repair of dysfunctional BM EPCs. Our preliminary data indicate that the use of TGF-β signaling inhibitors (such as luspatercept) might be a potential therapeutic strategy for patients with AA, which provides a rationale for further clinical trials in patients with AA to validate our preliminary findings in the future.

## Supplementary information

Supplementary Information
