# Supplementary material for: TGF-β inhibition restores hematopoiesis and immune balance via bone marrow EPCs in aplastic anemia
Source: Exp Mol Med. 2025 Jun 30;57(6):1324–38. doi: 10.1038/s12276-025-01483-4 (PMC12229535; doi:10.1038/s12276-025-01483-4)
Supplement: Supplementary file 1 — Supplementary Information [file 12276_2025_1483_MOESM1_ESM.pdf]

Supplementary Materials

TGF-β inhibition restores hematopoiesis and immune balance via bone marrow EPCs in aplastic anemia

Authors: Xin-Yan Zhang[1#], Li-Ping Guo[1#], Ya-Zhe Wang[1], Jin-Song Jia[1], Mi Liang[1], Meng-Zhu Shen[1], Zhen-Kun Wang[1], Zhi-Wei Zhang[1], Chen-Yuan Li[1], Zhong-Shi Lyu[1], Tong Xing[1, 2], Yuan-Yuan Zhang[1], Xiao-Jun Huang[1, 2, 3]*, Yuan Kong[1]*

[#] Xin-Yan Zhang and Li-Ping Guo are co-first authors.

***Xiao-Jun Huang and Yuan Kong are co-corresponding authors.

Correspondence: Yuan Kong**:** successky@163.com

Xiao-Jun Huang: huangxiaojun@bjmu.edu.cn

Supplementary Materials

Supplementary Table 1. Characteristics of AA patients and NCs

Characteristics	AA (N = 15)	NC (N = 15)	*P*-value*
**Physical variables**			
Age (years)[a]	54 (21-61)	53 (24-64)	0.89
Gender, female vs. male	8/7	6/9	0.46
**Blood cell counts**			
WBC count ($\times10^9$/L) [a]	3.19 (1.84-5.99)	6.63 (4.73-9.57)	< 0.0001
Neutrophil ($\times10^9$/L) [a]	1.33 (0.53-2.59)	3.97 (2.8-3.07)	< 0.0001
Lymphocyte ($\times10^9$/L) [a]	1.51 (0.66-2.90)	2.14 (1.29-3.19)	0.0064
Monocyte ($\times10^9$/L) [a]	0.27 (0.07-0.57)	0.35 (0.19-0.66)	0.10
RBC ($\times10^{12}$/L) [a]	2.93 (1.47-4.78)	4.92 (4.23-5.45)	< 0.0001
Hemoglobin (g/L) [a]	101.5 (56-169)	147.9 (117-172)	< 0.0001
Platelet ($\times10^9$/L) [a]	67 (6-151)	236.5 (149-377)	< 0.0001
Eosinophil ($\times10^9$/L) [a]	0.02 (0.00-0.06)	0.13 (0.04-0.41)	0.0005
Basophil ($\times10^9$/L) [a]	0.01 (0.00-0.03)	0.04 (0.01-0.10)	0.0003

[a] Data are reported as median(range).

* *P*-values present the comparison between the AA patients and NC groups.

Abbreviations: AA, aplastic anemia; NC, normal control; WBC, white blood cell; RBC, red blood cell.

Supplementary Table 2. The primer sequences of genes used for qRT-PCR

Gene	Forward primer sequence (5'-3')	Reverse primer sequence (5'-3')
18S	GTAACCCGTTGAACCCCATT	CCATCCAATCGGTAGTAGCG
FLT3LG	CGCTTCGTCCAGACCAACATCTC	GGGTGGCAGGGTTGAGGAGTC
KIT	GCGTTCTGCTCCTACTGCTTCG	TGGATGGATGGTGGAGACGGTTC
FLT3	GCAATCATAAGCACCAGCCAGGAG	TTCTGCGAGCACTTGAGGTTTCC
IL11RA	GCCAAGCAGCCGACTATGAGAAC	CCTGTGGATGGACTCCTCCTCTG

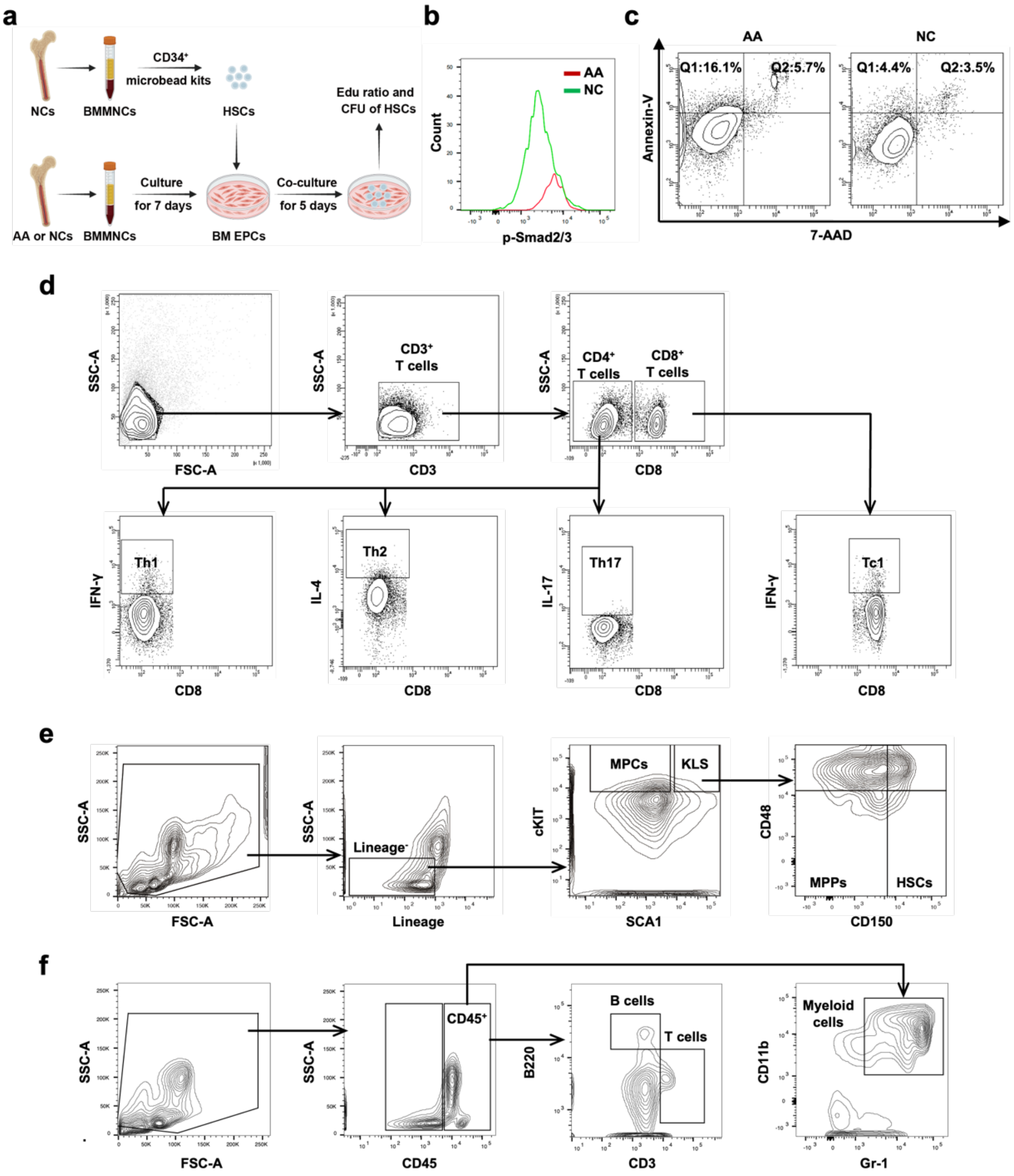

Supplementary Fig. 1. The schematic diagram of BM HSC-EPC co-culture assay and gating strategies for flow cytometry.

(a) The schematic diagram of BM HSC-EPC coculture assay. The EdU ratio and CFU efficiencies of HSCs were detected after direct-coculture for 5 days. Gating strategies for quantification of **(b)** phosphor-Smad2/3(pSmad2/3) level(MFI) of AA EPCs and NC EPCs, **(c)** the representative apoptosis ratios of BM EPCs from AA patients and NCs, which includes the total ratios of early

apoptosis cells (Q1: AnnexinV^{+}7-AAD^{-}) and late apoptosis cells (Q2: AnnexinV^{+}7-AAD^{+}), **(d)** BM Th1, Th2, and Th17 cells and Tc1 after cocultured with NC EPCs, **(e)** myeloid progenitor cells (MPCs, lineage^{-}cKIT^{+}SCA1^{-}), KLS (cKIT^{+}lineage^{-}SCA1^{+}) cells, multipotent progenitors (MPPs, lineage^{-}SCA1^{+}cKIT^{+}CD150^{-}CD48^{-}) and HSCs (lineage^{-}cKIT^{+}SCA1^{+}CD150^{+}CD48^{-}) in murine BM, and **(f)** myeloid cells (CD45^{+}Gr-1^{+}), B cells (CD45^{+}CD3^{-}B220^{+}) and T cells (CD45^{+}CD3^{+}B220^{-}) in murine BM by flow cytometry.
